# Early Differentiation of Human CD11c^+^NK Cells with *γδ* T Cell Activation Properties Is Promoted by Dialyzable Leukocyte Extracts

**DOI:** 10.1155/2016/4097642

**Published:** 2016-10-25

**Authors:** Dalia Ramírez-Ramírez, Eduardo Vadillo, Lourdes Andrea Arriaga-Pizano, Héctor Mayani, Sergio Estrada-Parra, Marco Antonio Velasco-Velázquez, Sonia Mayra Pérez-Tapia, Rosana Pelayo

**Affiliations:** ^1^Oncology Research Unit, Oncology Hospital, Mexican Institute for Social Security, Avenida Cuauhtémoc 330, Colonia Doctores, 06720 Mexico City, Mexico; ^2^Department of Immunology, National School of Biological Sciences (ENCB), National Polytechnic Institute (IPN), Carpio y Plan de Ayala s/n, Colonia Santo Tomás, 11340 Mexico City, Mexico; ^3^Immunochemistry Research Unit, Medical Specialties Hospital, Mexican Institute for Social Security, Avenida Cuauhtémoc 330, Colonia Doctores, 06720 Mexico City, Mexico; ^4^Unidad de Desarrollo e Investigación en Bioprocesos (UDIBI), National School of Biological Sciences (ENCB), National Polytechnic Institute (IPN), Carpio y Plan de Ayala s/n, Colonia Santo Tomás, 11340 Mexico City, Mexico; ^5^Unidad de Investigación, Desarrollo e Innovación Médica y Biotecnológica (UDIMEB), National School of Biological Sciences (ENCB), National Polytechnic Institute (IPN), Carpio y Plan de Ayala s/n, Colonia Santo Tomás, 11340 Mexico City, Mexico; ^6^Department of Pharmacology, School of Medicine, National Autonomous University of Mexico, Ciudad Universitaria, 04510 Mexico City, Mexico

## Abstract

Reconstitution of the hematopoietic system during immune responses and immunological and neoplastic diseases or upon transplantation depends on the emergent differentiation of hematopoietic stem/progenitor cells within the bone marrow. Although in the last decade the use of dialyzable leukocyte extracts (DLE) as supportive therapy in both infectious and malignant settings has increased, its activity on the earliest stages of human hematopoietic development remains poorly understood. Here, we have examined the ability of DLE to promote replenishment of functional lymphoid lineages from CD34^+^ cells. Our findings suggest that DLE increases their differentiation toward a conspicuous CD56^+^CD16^+^CD11c^+^ NK-like cell population endowed with properties such as IFNy production, tumor cell cytotoxicity, and the capability of inducing *γδ* T lymphocyte proliferation. Of note, long-term coculture controlled systems showed the bystander effect of DLE-stromal cells by providing NK progenitors with signals to overproduce this cell subset. Thus, by direct effect on progenitor cells and through activation and remodeling of the supporting hematopoietic microenvironment, DLE may contribute a robust innate immune response by promoting the emerging lymphopoiesis of functional CD11c^+^ NK cells in a partially TLR-related manner. Unraveling the identity and mechanisms of the involved DLE components may be fundamental to advance the NK cell-based therapy field.

## 1. Introduction

Emergency hematopoiesis defines the production of functional hematopoietic cells under nonhomeostatic, proinflammatory, or biologically stressed conditions [1–4]. Blood cell production is a tightly regulated process that, after birth and throughout life, starts in a conspicuous hematopoietic stem cell (HSC) subset residing within the bone marrow (BM). Our current understanding of how HSC early differentiation is governed by the microenvironment indicates that, besides the stromal cell components of the various hematopoietic niches, not only essential growth and differentiation factors but also microbes and their products can influence differentiation fate decisions [3, 5, 6]. Of note, emergency hematopoiesis is regulated at the stem and progenitor cell (HSPC) level, where conditions such as infection demand the expedited production and activation of innate immune cells to combat noxious extrinsic agents, and the resulting proinflammatory conditions can at the time regulate the earliest steps of the hematopoietic development in favor of the clearance of insulting cues and to further maintain homeostasis [1].

We have previously shown that pathogens and damaged tissue products and proinflammatory cytokines promote emergency hematopoiesis and alter patterns of early lymphoid differentiation in mouse and human [3–5, 7–10]. In mice, pathogen recognition through Toll like receptors (TLR) and the resulting cytokine release induce the expansion of HSC and instruct lineage differentiation fates so immediate innate cell development is guaranteed [6, 7]. In general, ligation of TLR2 and TLR4 on these seminal cells promotes redirection toward myeloid cell production, while the sole TLR9 stimulation of primitive common lymphoid progenitors (CLP) strikingly induces B cell differentiation blockage while development of dendritic cells (DC), plasmacytoid dendritic cells (pDC), and NK-related interferon killer dendritic cells (IKDC) is substantially enforced [5, 8]. In humans, most findings relate to strengthening of myeloid lineage cell production under emergent scenarios, whereas adjustments within the lymphoid branch of the hematopoiesis have been poorly addressed [2, 6]. According to what mouse research has shown, human multilymphoid progenitors (MLP) are capable of responding to TLR stimulation by producing dendritic cells, and our recent work suggests that primitive early lymphoid progenitor populations are also capable of microbial components discrimination through TLR, a mechanism that mostly facilitates their differentiation to innate lymphoid lineage cells. Of special interest, TLR9 ligation on adult BM progenitors promotes the quick development of NK lineage cells by using mechanisms that involve IL-15R upregulation [4, 9].

Thus, innate immune quick responses against viral threatening infections start in earlier developmental stages than we previously thought. Whether the actual TLR-emergent hematopoiesis contributes to innate immunity under pathological conditions and other biological stress settings, including malignant diseases, is a highly relevant topic under investigation [11]. Interestingly, not only conventional pathogen associated molecular patterns (PAMPs) but also the damage associated molecular patterns- (DAMPs-) like molecules can trigger innate immune sensors and PRR signals, including microRNAs, histones, fibronectin, and bacterial second messengers like di-GMP (reviewed in [3, 12]). Even though efficient therapeutic agents have been developed that improve infectious and malignant disease outcomes and increase the overall survival rates, the adjuvant effect of molecules capable of remodeling hematopoietic pathways should be taken into consideration to change the prognosis of diseases. Thus, the possibility of having extensive means of replenishing innate cells opens additional venues for receptor-ligand axes of clinical significance.

Disruption of normal peripheral blood leukocytes results in the release of heterogeneous mixtures of peptides, among other complex molecules. Upon dialysis, the enriched mixture of low-molecular-weight polar and hydrophilic peptides (<10 kDa), named dialyzable leukocyte extracts (DLE), has shown a number of therapeutic and adjuvant properties through modulation of immune responses [13, 14]. Although the precise molecular mechanisms underlying its positive experimental and clinical effects are currently unknown, critical signaling pathways for survival and cellular activation states, including Toll like receptor (TLR) and NF*κ*B, are apparently involved and often trigger the production of proinflammatory cytokines [14–18]. Of note, a recent investigation using* in vitro* controlled models of TLR-mediated proinflammation suggested the content of TLR-2 agonist ligands within DLE [15]. The exposure of human peripheral mononuclear cells to DLE induced the copious secretion of TNFalpha by the monocyte fraction. Whether this phenomenon is due to DAMPs or DAMP-like related peptides within DLE is still a matter in question.

Using an elegant mouse model of experimental tuberculosis, Fabre and colleagues demonstrated that the administration of DLE (formerly denominated transfer factor) evokes an efficient reconstitution of the cell-mediated immunity, concomitant with a substantial production of IFNalpha, IL-2, and iNOS, an immune protective profile provoking inhibition of bacterial proliferation [19, 20].

In contrast, inflammatory injuries of human ocular tissues where limbal epithelial stem cells giving rise to corneal epithelium are compromised have been shown to respond to DLE treatment by downregulating the secretion of IL-8 and IL-6 [18]. The same is true for atopic dermatitis, where DLE contribute the decrease of inflammatory cells and the severity of the disease [21]. Then, despite the proinflammatory potential induction of DLE, the net balance—induction or suppression—may depend on additional biological settings of the damaged tissues, where the dose of DLE would be absolutely crucial to get a beneficial result [20].

Resolution of herpes virus infections is remarkably benefited from the adjuvant effect of DLE treatment [13, 16]. In fact, a herpes murine model has become a powerful biological assay to test the functional activity of DLE [22]. Of special interest, a direct effect on viral replication or infected target cell viability could not been recorded. Instead, its protective effects correlated with serum cytokine levels and, most likely, with changes in the cellular immune system. Accordingly, as cell-mediated immunity plays a central role in controlling viral-infected and tumor cells, and the capability of DLE of strengthening the cellular immunity has been suggested from several studies, DLE is considered as a presumptive instrument with adjuvant potential for treating virally induced cancer [13]. Moreover, a growing list of viral, parasitic, or fungal infections, as well as acute and chronic diseases, including immunodeficiencies, malignancies, allergies, and autoimmune disorders, seems to favorably respond to DLE.

However, a more comprehensive understanding of its biological mechanisms is yet needed and will be benefited from less heterogeneous and highly controlled extracts. DLE Transferon, a blood product licensed for clinical use, has been shown to exhibit batch-to-batch- reproducibility and relatively high homogeneity when ultra-performance liquid chromatography (SE-UPLC) is used to characterize its content [14]. Due to the putative TLR agonist elements within DLE and their biological capability of inducing proinflammatory microenvironments, here we sought to examine the DLE's contribution to innate immune replenishment through emergent hematopoiesis. By using* in vitro* functional assays and controlled early differentiation culture systems, our findings define a powerful route to promote development of a unique CD11c^+^ NK cell subset with immune-surveillance capacity.

## 2. Materials and Methods

### 2.1. Sample Collection and Progenitor Cells Isolation

Umbilical cord blood (UCB) samples were obtained from normal full-term neonates upon mothers' written informed consent, while adult bone marrow (ABM) was collected from healthy adult donors who entered orthopedic surgery and according to institutional guidelines. Mononuclear cells (MNCs) were prepared by Ficoll-Paque Plus (GE Healthcare Bioscience) gradient centrifugation and preserved at −80°C until use. All procedures were approved by the Ethics and Scientific Committee of Health Research at IMSS (R-2006-3602-16).

### 2.2. Isolation of Cell Populations and Flow Cytometry

CD34^+^ cells from ABM and UCB were enriched using the Human CD34 Progenitor Cell Isolation Kit (Miltenyi Biotec) according to manufacturer instructions and our previous reports [9]. After staining with PE-conjugated antilineage markers (CD3, CD8, TCR, CD56, CD14, CD11b, CD20, CD19, and CD235a), anti-CD34-APC, anti-CD38-FITC, and anti-CD45RA-PE-TxR conjugated antibodies, primitive cell populations were highly purified by multicolor flow cytometry using a FACSAria sorter (BD Biosciences). Hematopoietic stem cells (HSC) were separated as Lin^−^CD34^+^CD38^−^CD45RA^−^, while multipotent progenitors (MPP) were sorted as Lin^−^CD34^+^CD38^+^CD45RA^−^, and early lymphoid progenitors (ELP) as Lin^−^CD34^+^CD38^+^CD45RA^+^, as described [9]. Upon harvesting from culture, anti-CD56-PE, anti-CD11c-FITC, and anti-CD16-APC (BD Biosciences) were used to evaluate innate cell production in lymphoid lineage conditions. NK cells were identified by flow cytometry in a FACSCanto II equipment (BD Biosciences) as CD56^+^CD11c^+^ or CD56^+^CD16^+^CD11c^+^ cells, while dendritic cells (DC) were detected as CD56^−^CD11c^+^. Analysis of flow cytometry data was performed using the FlowJo 10 software (TreeStar Inc., USA).

### 2.3. Dialyzable Leukocyte Extracts (DLE Transferon) Stimulation

DLE Transferon is manufactured by UDIMEB at GMP facilities in the National School of Biological Sciences, National Polytechnic Institute (IPN), as described [14, 15]. Transferon is registered by Mexican Health Authorities as a drug and commercialized nationally. Briefly, DLE were prepared from packed blood peripheral leukocytes from healthy donors. Cells were disrupted by 5 cycles of freezing and thawing followed by dialysis against water using Spectra/Pore membranes with a “cut-off” of 12 KDa (Spectrum Labs, USA). The quality control of Transferon comprised (A) endotoxin content, quantified using the Endosafe-Portable Test (Charles River Laboratories, Charleston, SC, USA) according to the manufacturer's instructions (the specification for endotoxin was established in Mexican Pharmacopeia, Section MGA-0316 (≤4.0 EU/mL)); (B) microbiological tests, according to Mexican Pharmacopeia, Section MGA-0571; and (C) physicochemical characterization by a validated ultra-performance liquid chromatography (UPLC) method that analyzes molecular weights and the time of retention of the main peaks compared with those of an internal batch pattern. Peptide content per final dose was measured by bicinchoninic acid (BCA) method using the Pierce BCA kit (Thermo Fisher Scientific, Waltham MA, USA) according to the manufacturer's instructions [22]. Batch-to-batch reproducibility is consistently analyzed by SE-UPLC chromatographic profiles, while IFN*γ* production from DLE-stimulated Jurkat cells is quantified as a biological activity test [14].

Enriched CD34^+^ cells from UCB and ABM were cultured for 24 hours in *α*-MEM medium supplemented with 10% fetal bovine serum, 100 U/mL penicillin, and 100 mg/mL streptomycin. DLE Transferon was used for cell culture stimulation at 5 *μ*g/mL.

### 2.4. Stromal Cell Cocultures

MS-5 stromal cells were grown in presence or not of DLE Transferon 24 hours before coculturing with CD34^+^ HSPC. On the other hand, and upon 24 hours of DLE prestimulation, HSPC (including the whole enriched CD34^+^ fraction or Lin^−^CD34^+^CD38^−^CD45RA^−^ HSC, Lin^−^CD34^+^CD38^+^CD45RA^−^ MPP, or Lin^−^CD34^+^CD38^+^CD45RA^+^ ELP) were centrifuged to remove the medium and cocultured in the presence of MS-5 stromal cells for 30 more days. Cocultures were performed using *α*-modified essential medium (*α*-MEM) (GIBCO) supplemented with 10% fetal bovine serum and 100 U/mL penicillin and 100 mg/mL streptomycin. Lymphoid lineage cytokines and growth factors were contained throughout coculture: 1 ng/mL Flt3-L (FL), 2 ng/mL SCF, 5 ng/mL IL-7, and 10 ng/mL IL-15 (Preprotech). Coculture systems were incubated at 37°C in a humidified atmosphere of 5% CO_2_.

### 2.5. BrdU Incorporation Assay

Enriched CD34^+^ cells from UCB and ABM were cultured for 72 hours with 5 *μ*g/mL of DLE Transferon and bromo-2′-deoxyuridine (BrdU) to a final concentration of 10 *μ*M, in *α*-MEM serum-free medium supplemented with lymphoid cytokines. After stimulation, cells were stained for the identification of CD34^+^ cell progenitors by multicolor flow cytometry followed by intracellular staining with a monoclonal antibody to BrdU according to an established protocol (BrdU flow kit, BD Biosciences). Cells were analyzed on a FACSCanto II flow cytometer (BD Biosciences).

### 2.6. Inhibition of TLR Signaling Pathway

UCB CD34^+^ cells were pretreated with 20 *μ*M of MyD88 control or inhibitory peptides (Imgenex IMG-200501) for two hours, followed by 2x washing and staining with 5 *μ*M Cell Trace Violet (CTV) dye. DLE Transferon was included or not at 5 *μ*g/mL in a 72 h culture. Harvested cells were analyzed by flow cytometry for cell division numbers. The dilution of fluorescence intensity was estimated using the application for cell proliferation from the FlowJo 7.6.2 software.

To investigate the influence of TLR signals in the DLE-mediated NK-like cell differentiation, UCB CD34^+^ cells were pretreated with 20 *μ*M of MyD88 control or inhibitory peptides (Imgenex IMG-200501) for 24 hours, followed by 2x washing and coculturing on MS-5 stromal cells for 15 more days. Cocultures were performed using *α*-modified essential medium (*α*-MEM) (GIBCO) supplemented with 10% fetal bovine serum and 100 U/mL penicillin and 100 mg/mL streptomycin (as described above in Section 2.4).

### 2.7. Cytotoxicity Assay

Natural killer cytolytic activity was evaluated using a fluorescence-based assay, as described [9, 23]. This assay uses the dye carboxyfluorescein succinimidyl ester (CFSE) to distinguish target from effector cells, and the DNA intercalating dye 7-aminoactinomycin D (7-AAD) (BD Pharmingen) for dead/live cells distinction. Briefly, the myeloid leukemia K562 cell line (ATCC) was kept in log phase growth in RPMI 1640 + GlutaMAX supplemented with 10% of fetal calf serum (FCS). K562 cells were stained with 5 *μ*M carboxyfluorescein succinimidyl ester (CFSE).* In vitro* differentiated NK cells from DLE-cultures were enumerated by flow cytometry and cocultured with CFSE-labeled K562 cells, according to an effector: target ratio curve. A relation of 10 CD56+ cells per each K562 cell was used. IL-2 (40 ng/mL) (Preprotech) was added to induce NK cell activation, followed by 4 hours of incubation at 37°C. Cells were washed and 7-AAD (BD Pharmingen) was incorporated into the cell suspension. Multiparametric flow cytometry (FacsCanto II, BD Biosciences) was conducted to determine functionality.

### 2.8. Intracellular Detection of IFN-Gamma

To investigate the capability of DLE-newly derived NK-like cells of producing IFN-gamma (IFN*γ*) upon stimulation, an IFN*γ* assay was performed. After 30-day coculture, the produced cells were stimulated with IL-12 (10 ng/mL) and IL-18 (50 ng/mL) for 12 hours. NK-like cells were harvested and Brefeldin A (BFA, 10 *μ*g/mL) was added for 4 hours to inhibit protein intracellular transport. After BFA blockage, cells were carefully washed, followed by incubation with anti-CD56-APC, anti-CD11c-FITC (BD Biosciences), and anti-IFN*γ*-PE (Biolegend) conjugated antibodies to perform extra- and intracellular staining, respectively. A FACSCanto II equipment was used for multiparametric flow cytometric analysis.

### 2.9. Proliferation Induction Assay of *γδ* T Lymphocytes

Gammadelta T peripheral blood lymphocytes (TCR*γδ*-1) from healthy donors were enriched by the *γδ* T Lymphocyte Isolation Kit (MiltenyiBiotec, IgG1 clone 11F2) according to manufacturer instructions, followed by staining with 5 *μ*M Cell Trace Violet (CTV). Simultaneously, newly differentiated CD11c^+^CD56^+^ NK cells from DLE-stimulated 30-day cocultures were harvested and purified by flow cytometry sorting in BD FACSAria equipment. Postsort cell purity was investigated by using the anti-human TCR *γ*/*δ* mouse IgG1 antibody clone B1. The purified *γδ* T cells were then cocultured with the NK cell population of interest, at a ratio of 2 : 1 CD56^+^CD11c^+^ NK : *γδ* T lymphocyte. Supplemented *α*-MEM medium with 10% fetal bovine serum, 100 U/mL penicillin, and 100 mg/mL streptomycin was used. *γδ* T cells proliferation was assessed at 72 hours by dilution of CTV (within the Pacific Blue channel).

### 2.10. Statistics

The Prism software, version 5.01 (GraphPad, USA), was used for statistical analysis. Comparisons between groups were performed with either the unpaired *t*-test or the one-way ANOVA. *P* values were 2-tailed and considered significant if less than 0.05.

## 3. Results and Discussion

### 3.1. Human Hematopoietic Stem/Progenitors Cells Are Promoted by Dialyzable Leukocyte Extracts (DLE) to Differentiate into CD11c^+^ NK-Like Cells

**Figure 1:**
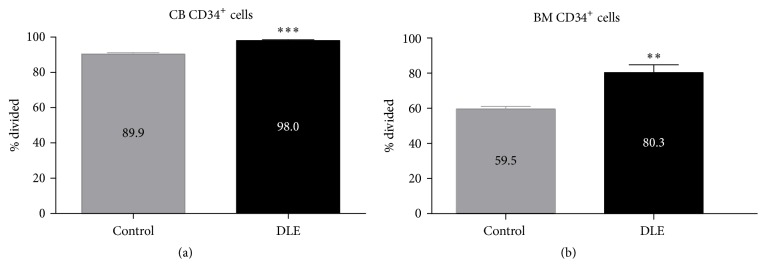
Dialyzable leukocyte extracts (DLE) promote proliferation of primitive hematopoietic CD34^+^ cells from neonatal and adult sources. CD34^+^ cells from adult bone marrow (ABM) and umbilical cord blood (UCB) were enriched and cultured for 72 hours with DLE Transferon and BrdU. Cells were stained for the identification of CD34^+^ cell progenitors followed by intracellular staining of BrdU. Multiparametric flow cytometry was performed in a FACSCanto II and the cell frequencies of BrdU^+^ cells calculated. Data are representative of 3 independent experiments. ^*∗*^ *P* < 0.05; ^*∗∗*^ *P* < 0.01.

Our previous research on mouse and human early hematopoietic fate redirection in response to extrinsic PRR agonists prompted us to investigate whether this phenomenon may occur upon exposure to DLE Transferon. To first determine sensitivity of primitive populations to DLE components, we performed BrdU incorporation analyses on CD34^+^ cell fractions from cord blood and adult bone marrow. Notably, we recorded significant increases of proliferative cell frequencies from both sources after 72 h of DLE stimulation (Figure 1). Furthermore, bone marrow cell stimulation under lymphoid or myeloid culture conditions revealed that adult lymphoid progenitors preferentially expand in response to DLE (Supplemental Figure  1 in Supplementary Material available online at http://dx.doi.org/10.1155/2016/4097642).

**Figure 2:**
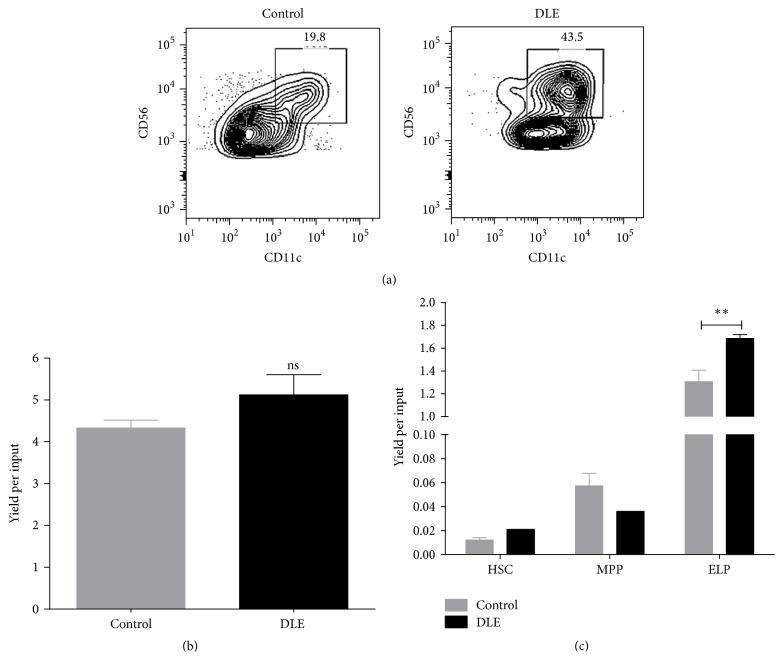
CD56^+^CD11c^+^ NK-like cells expeditiously develop from early lymphoid progenitors upon DLE stimulation. Upon 24 hours of DLE Transferon stimulation, the whole fraction of CD34^+^ cells (a and b) or purified primitive cells, including hematopoietic stem cells (HSC), multipotent progenitors (MPP), and early lymphoid progenitors (ELP) (a), were cocultured with MS-5 stromal cells for 30 days under lymphoid lineage conditions. CD56^+^CD11c^+^ NK-like cell production was investigated by flow cytometry and reported as cell frequencies (a) or yield per input progenitor (b and c). For comparison, unstimulated cultures were considered as control conditions. DLE, dialyzable leukocyte extracts Transferon. ^*∗∗*^ *P* < 0.01.

Natural Killer (NK) cells constitute a granular innate immune cell type comprising 3–20% of the human peripheral blood mononuclear cell (PBMC) lymphocyte fraction, which function as major players in innate responses by producing cytokines and chemokines and exerting cytolytic activity against virus-infected or tumor cells [24–26]. Here, DLE stimulation of CB CD34^+^ cells rapidly influenced early cell fate decisions and accelerated the differentiation toward a special population of CD56^+^CD11c^+^ NK cell (Figure 2). Although final yields per input progenitor were not indicative of higher net production when the full CD34 compartment is considered, substantially discrepant CD56^high^CD11c^+^ cell frequencies suggest the speeding up of such specialized cells (Figures 2(a) and 2(b)). In line with the observations in the context of TLR signaling [9], cells harboring early lymphoid progenitor phenotype were the major producers of DLE-newly differentiated NK cells (Figure 2(c)).

The identity of CD11c^+^ NK cells has been a matter in question and a debate point for years. The various studies from DeMatteo have indicated that CD11c expression defines a distinct subset of murine liver NK cells that respond to adenoviral infection by producing IFN-gamma in a TLR9-IL-12-IL-18-dependent way [27]. Moreover, the same NK dendritic cells (NKDC) phenotype was found to be multifunctional cells with pleiotropic functions, including the IFN*γ*-mediated response against bacterial infections and malignant processes, and the migration to central nervous system for viral clearance [28–32]. Belonging to the Hardy Fraction A of the mouse B cell developmental pathway, the discrete B220^+^CD11c^+^NK1.1^+^ NK subset seemed to meet the biological properties of NKDC, that is, dependency on IL-15 and ability of producing highest amounts of IFN*γ* [33]. Of relevance, two simultaneous reports recently revealed the “hybrid” phenotypic and functional characteristics of DC and NK within a novel subset of interferon-producing killer dendritic cell (IKDC) population, endowed with strong cytotoxic and antitumor activities, and antigen presenting competence [34–37]. IKDCs share some properties with other innate lymphoid cells, but their development is unique and is most efficiently generated from L-selectin^+^ BM progenitors, while common lymphoid progenitors (CLP) are the most effective source of conventional NK cells [36]. Even though a IKDC subset has not been formally described in humans, expression of CD11c in NK lineage cells is induced by combination of IL-15 and inflammatory cytokines and distinguishes a conspicuous cell subtype participating in immune regulation of chronic diseases [38].

Our studies were then extended to CD11c^+^ dendritic cells. Starting from the HSC fraction, DLE stimulation could efficiently trigger their production (Supplemental Figure 2), emphasizing its potential to aim at innate responses but suggesting the divergent origin of DLE-derived NK and DC. Accordingly, animals treated with DLE have showed increased NK cell frequencies and numbers, among other hematopoietic cells [19, 20].

Due to their fundamental position in viral and tumor immune-surveillance, extensive efforts have been achieved to define experimental conditions to expand NK cells for adoptive immunotherapy [24]. Our data suggest a preliminary strategy to produce high numbers of these cells and, most of all, to robust the* in vivo* production by remodeling central hematopoiesis. Of note, inhibition of MyD88 adaptor molecule reduced proliferation and NK-differentiation potentials of DLE-stimulated CD34^+^ cells, suggesting the partial contribution of TLR signaling pathway in this emergent phenomenon (Supplemental Figure 3).

### 3.2. DLE-Associated Microenvironmental Activation Contributes the Emergent Production of CD11c^+^ NK Cells

Despite the wealth of information relevant to transcription factors- and phenotype-associated biological functions of NK lineage cells, we still need to understand the role of environmental niches in their early differentiation under steady-state and pathological conditions [24]. It is well known that they are generated and perform their critical functions in the context of hematopoietic niches within the bone marrow [24, 39] or in extramedullar tissues, respectively. Indeed, besides the identified developmental niches, an immune response niche, an inflammatory or stress niche, a tumor niche, and a pregnancy niche have been proposed to regulate the biology of NK cells. Such niches are presumably controlled by nonhematopoietic cells providing signals triggered from adhesion molecules, chemokines, and cytokines/growth factors [39–41]. Generation of NK cells requires IL-15 and Fms-like tyrosine kinase 3 ligand, but not IL-7. Strikingly, the CXCR4/CXCL12 axis conformed by CXCL12-abundant reticular (CAR) mesenchymal cells and NK precursors might function as the major niche element and is crucial for NK development within BM [39, 41]. Moreover, production of IL-15 is the highest in CAR cells.

**Figure 3:**
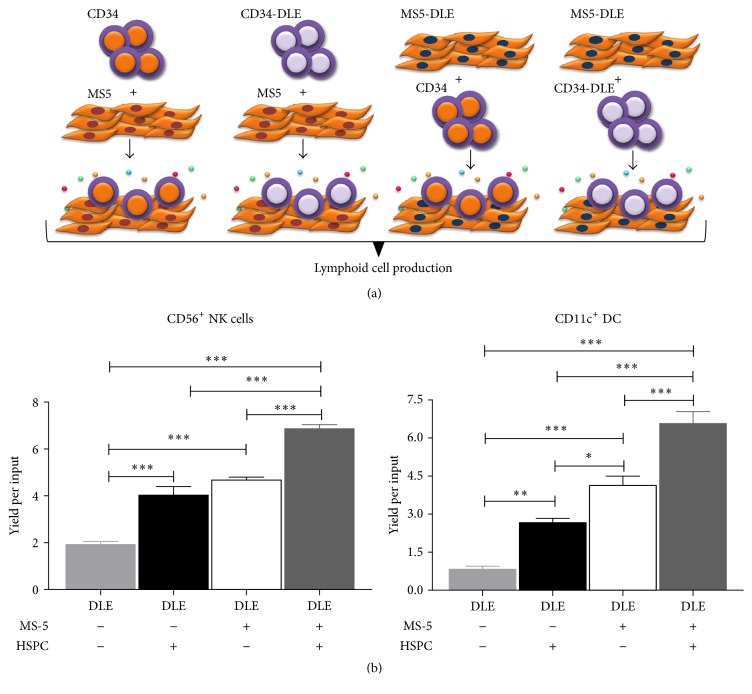
Stromal cell activation by DLE increases the emergent innate immune cell differentiation. MS-5 stromal cells were exposed or not to DLE Transferon 24 hours before coculturing with CD34^+^ hematopoietic stem and progenitor cells (HSPC) that were pretreated 24 hours with DLE Transferon (a). All conditions were placed in lymphoid lineage cocultures for 30 days followed by flow cytometry analyses of CD56^+^ NK cells and CD11c^+^ DC production. Yields per input progenitor were tabulated to show the positive contribution of activated stromal cells to innate differentiation (b). DLE, dialyzable leukocyte extracts Transferon. ^*∗*^ *P* < 0.05; ^*∗∗*^ *P* < 0.01; ^*∗∗∗*^ *P* < 0.001.

Our data confirm that microenvironment cues affect the development and biological roles of distinct NK subsets and suggest a substantial positive effect of DLE on stromal cell activation that, in turn, may promote CD11c^+^NK and DC expeditious differentiation (Figure 3). The additive differentiation effect by stromal cells may result from the action of cytokines and NK growth factors. Certainly, a multiplex type assay has shown the differential production of lymphoid factors and VEGF (our preliminary unpublished observations).

Two main subtypes of human NK cells are recognized: CD56^low^CD16^+^ and CD56^high^CD16^+/−^. While the abundant peripheral CD56^low^CD16^+^ NK cells are phenotypically classified as the most cytotoxic ones, the less copious CD56^high^CD16^+/−^ subset has been shown to be endowed with noticeable cytokine production capability. Their activation is mediated by the net balance between activating and inhibitory receptor engagement signals and in response to cytokines such as IL-2, IL-12, IL-15, and IL-18 [25]. Among activating molecules displayed by the CD56^low^CD16^+^ NK cells, CD16 plays a critical role in targeting antibody Fc-bound cells and executing antibody dependent cell-mediated cytotoxicity (ADCC). Expression of granzyme B and perforin is also high compared to the CD56^high^ counterpart. Predominantly in tissues and secondary lymphoid organs, the CD56^high^CD16^+/−^ subset is responsible for production of IFN*γ*, TNF*α*, GM-CSF, and RANTES after activation.

The recent reevaluation of functional properties of both cell categories has changed the binary paradigm of NK biology, providing clear evidence of the rapid and simultaneous effector activities exerted by the same CD56^low^CD16^+^ NK cell subset [25]. Thus, a prompt and abundant IFN*γ* production overlaps with their cytolytic ability and may lead to amplification of macrophage and DC-mediated responses.

**Figure 4:**
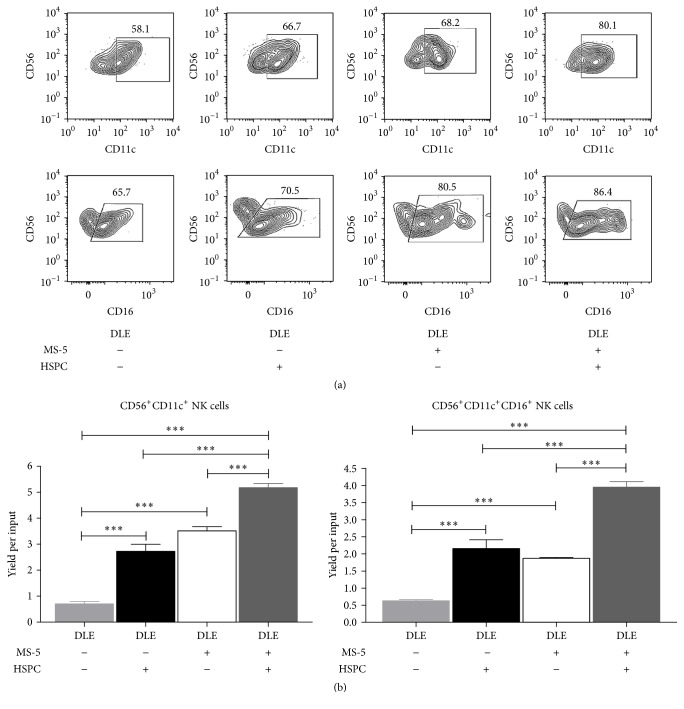
Preferential production of functional CD11c^+^CD16^+^ NK-like cells promoted by hematopoietic-stromal cell communication responding to DLE. MS-5 stromal cells were exposed or not to DLE Transferon 24 hours before coculturing with DLE Transferon-pretreated CD34^+^ hematopoietic stem and progenitor cells (HSPC), as described in Figure 3(a). All conditions were placed in lymphoid lineage cocultures for 30 days followed by flow cytometry analyses, where the indicated gates were used to discriminate CD56^+^CD11c^+^ and CD56^+^CD16^+^CD11c^+^NK cell frequencies (a). Yields per input progenitor were tabulated to record the significant variations by stromal or hematopoietic cells exposure to DLE Transferon (b). ^*∗∗∗*^ *P* < 0.001.

Interestingly, we have now found that most DLE-newly produced cells expressed CD16 and that microenvironmental prestimulation induces the density of this molecule (Figure 4).

### 3.3. DLE-Derived CD11c^+^ NK Cells May Function As Antiviral and Antitumoral Cell Mediators

**Figure 5:**
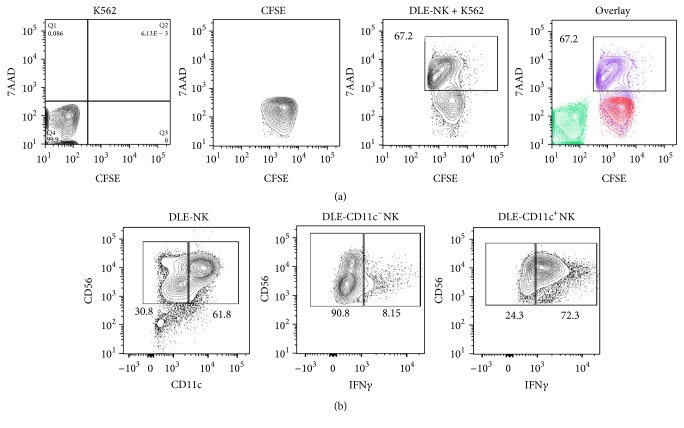
Effector functions of CD11c^+^ NK cells differentiated from DLE-induced lymphoid progenitor cells include tumor cytotoxicity and IFN-gamma production. NK cytotoxicity function was evaluated on CFSE+ K562 tumor cells as target of NK-like cells derived from DLE stimulation (DLE-NK) (a). We used 7-aminoactinomycin D (7-AAD) to evidence the frequency of CFSE^+^7-AAD^+^ killed target cells. Production of IFN-gamma by CD11c^−^CD56^+^ and CD11c^+^CD56^+^ NK cells was detected by intracellular staining (b).

The functional competence of NK cells produced from DLE-stimulated lymphoid progenitors was tested, demonstrating a very efficient cytolytic potential when a nonradioactive method was used (Figure 5(a)). We also observed a copious production of IFN*γ* only from the specialized CD11c^+^ NK population (Figure 5(b)), which is highly relevant to antitumoral and antimicrobial immune protection as this proinflammatory cytokine is pivotal in initiating Th1 immune responses against pathogens and tumors [30, 31].

Thus, these data suggest that early progenitors are promoted to differentiate toward both cytotoxic and regulatory cells in response to DLE. As mentioned before, the CD16^+^ NK subset may contain both biological properties [25].

**Figure 6:**
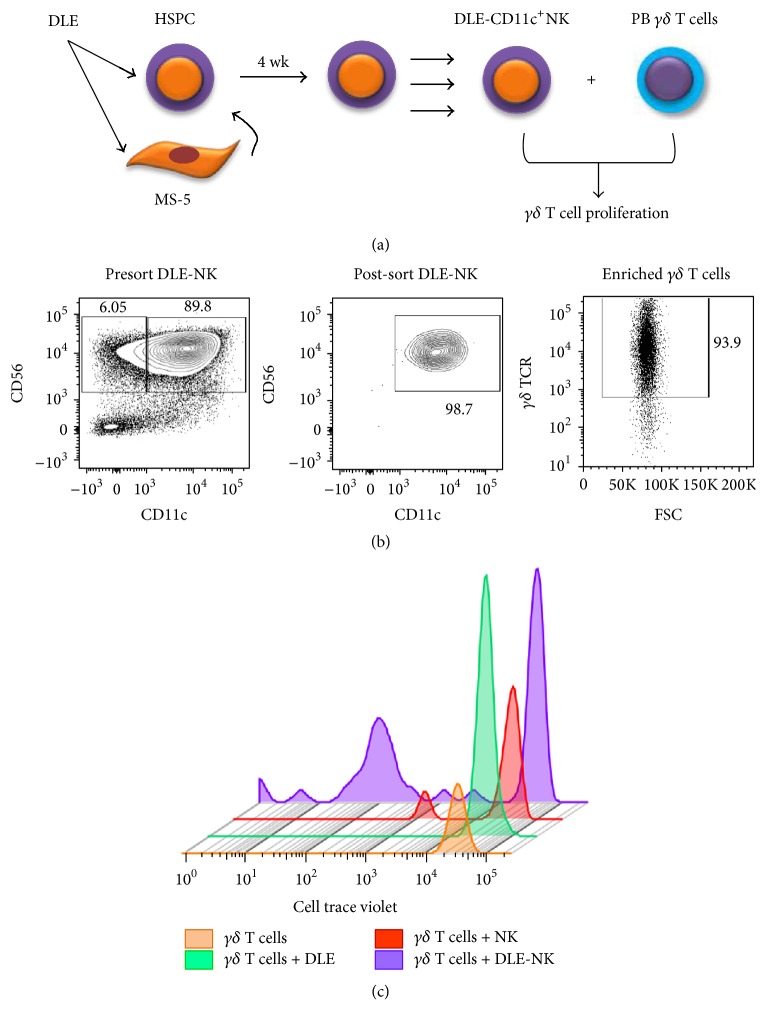
Activation of *γδ* T cells as a result of DLE-associated CD11c^+^ NK cell emergent production. *γδ* T cells from healthy donors were cocultured with the DLE-derived CD11c^+^ NK cell at a ratio of 2 : 1 CD56^+^CD11c^+^ NK : *γδ* T cell (a). Both NK and *γδ* T cell populations were highly purified by flow cytometry sorting before setting the proliferation assay (b). *γδ* T lymphocyte proliferation was assessed at 72 hours by dilution of Cell Trace Violet (CTV) dye (c).

**Figure 7:**
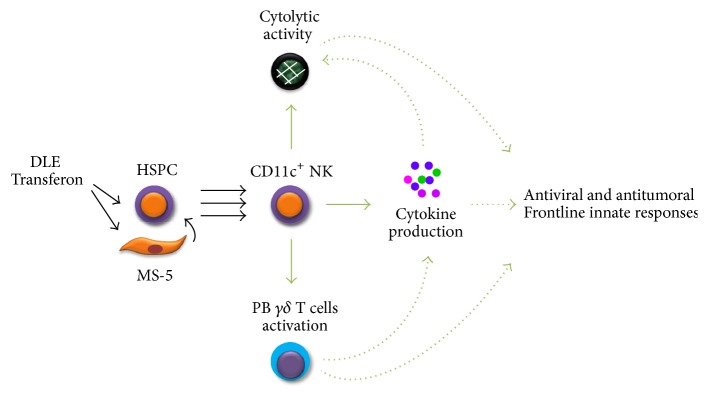
Emergent CD11c^+^ NK cell production contributing the adjuvant immune-surveillance effects of DLE Transferon: a proposed model. Dialyzable leukocyte extracts Transferon promote the early differentiation of functional CD11c^+^ NK cells endowed with the capabilities of tumor cell cytotoxicity, IFNy production, and *γδ* T lymphocyte proliferation induction, which may in turn contribute to innate immune responses against virus-infected or tumor cells. The potential involved mechanisms require further investigation.

Recently, CD56^hi^CD11c^+^ human NK-like cells were shown to induce the proliferation of *γδ* T cells in an IL-18 dependent manner [42, 43]. Because V*γ*2V*δ*2 T lymphocytes are key components of the innate immune system that function against infections and human tumor cells, we explore the faculty of DLE-NK cells of activating them. Of note, dilution of the dye CTV was significantly apparent when *γδ* T cells were cultured with CD11c^+^NK that are produced upon stimulation with DLE. Surprisingly, neither unstimulated *γδ* T cells nor DLE-directly stimulated *γδ* T cells got activated (Figures 6 and 7). Activation of *γδ* T cells can be achieved by a number of microorganisms and phosphoantigens and does not require antigen processing, but some atypical costimulatory molecules such as NKG2D play crucial roles in activating them to kill neoplastic or pathogen-infected cells [44].

These innate cells function through a variety of mechanisms, including secretion of cytokines and chemokines, dendritic cell activation, macrophage recruitment, cytolytic activity, and antigen presentation [45]. Their contribution to immune-surveillance in a major histocompatibility complex- (MHC-) unrestricted way, by their capability of producing IFN*γ* and TNF*α* and via an NK-like pathway, has positioned them as attractive targets for immunotherapy strategies [45–48].

Taken together, our study provides a rational explanation for the adjuvant contribution of DLE to the observed effective immune responses against viral and malignant diseases. DLE could play an inductor role in the NK lineage emergent hematopoiesis, suggesting the need of elucidating central mechanisms that govern DLE-associated tissue regeneration and give an insight into novel cooperative drug activities.

## 4. Concluding Remarks

While remarkable progress has long been recorded in identifying targets for cellular-based immunotherapeutic strategies to improve acute and chronic disease outcomes, it is becoming clear that definition of extrinsic factors promoting innate cell differentiation processes from the earliest developmental stages may change our vision of immunomodulation. Our findings suggest, for the first time, that, in response to dialyzable leukocyte extracts (DLE) Transferon, human stem and progenitor cells contribute to the emergent production of a special subset of innate CD11c^+^ NK cells. Of note, this DLE-derived NK cell population is endowed with properties such as IFN*γ* production, tumor cell cytotoxicity, and the capability of inducing *γδ* T lymphocyte proliferation that may, in turn, function as coadjuvant component of innate immune responses against virus-infected or tumor cells.

## Supplementary Material

Supplemental Figure 1 shows that ability of dialyzable leukocyte extracts (DLE) Transferon® of promoting proliferation of primitive lymphoid and myeloid progenitors can be extended to the adult hematopoietic source bone marrow. Supplemental Figure 2. As previously reported for TLR signalling in mice and human early progenitors, emergency hematopoiesis of CD56-CD11c^+^ dendritic lineages can be induced upon in vitro DLE stimulation. Supplemental Figure 3. To investigate the contribution of TLR signalling pathway activation in the DLE-dependent emergent differentiation of NK cells, we did inhibit the MyD88 adaptor function. A partial reduction of DLE promoting effect was recorded, suggesting that emergency NK differentiation results from multiple mechanisms.
