# Supplementary material for: Early Differentiation of Human CD11c+NK Cells with γδ T Cell Activation Properties Is Promoted by Dialyzable Leukocyte Extracts
Source: J Immunol Res. 2016 Oct 25;2016:4097642. doi: 10.1155/2016/4097642 (PMC5099461; doi:10.1155/2016/4097642)
Supplement: Supplementary file 1 — Supplemental Figure 1 shows that ability of dialyzable leukocyte extracts (DLE) Transferon® of promoting proliferation of primitive lymphoid and myeloid progenitors can be extended to the adult hematopoietic source bone marrow. Supplemental Figure 2. As previously reported for TLR signalling in mice and human early progenitors, emergency hematopoiesis of CD56-CD11c+ dendritic lineages can be induced upon in vitro DLE stimulation. Supplemental Figure 3. To investigate the contribution of TLR signalling pathway activation in the DLE-dependent emergent differentiation of NK cells, we did inhibit the MyD88 adaptor function. A partial reduction of DLE promoting effect was recorded, suggesting that emergency NK differentiation results from multiple mechanisms. [file 4097642.f1.docx]

**SUPPLEMENTAL FIGURES Ramirez et al.**


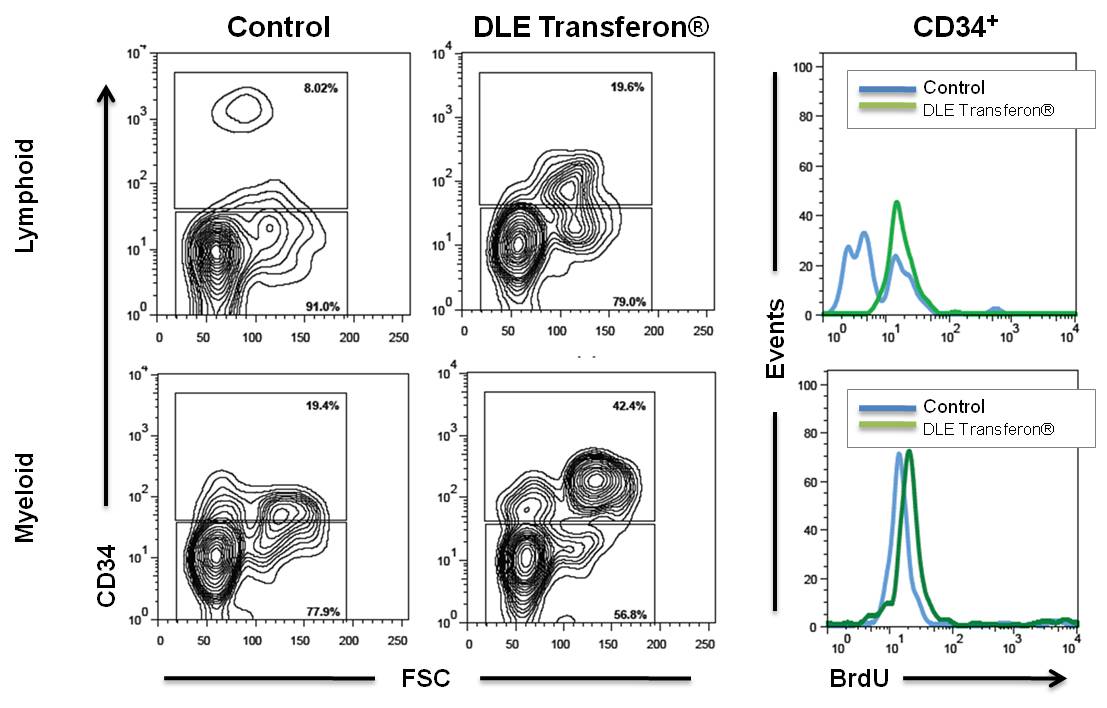


**Supplemental Figure 1.** **Dialyzable leukocyte extracts (DLE) Transferon® promote proliferation of lymphoid and myeloid progenitors from human adult bone marrow**. Mononuclear cells from adult bone marrow (ABM) were enriched, and cultured for 72 hours with DLE Transferon^®^ and BrdU under lymphoid or myeloid conditions. Cells were stained for the identification of CD34^+^ progenitors followed by intracellular staining of BrdU. Multiparametric flow cytometry was performed in a FACSCanto II and the cell frequencies of BrdU^+^ cells calculated.


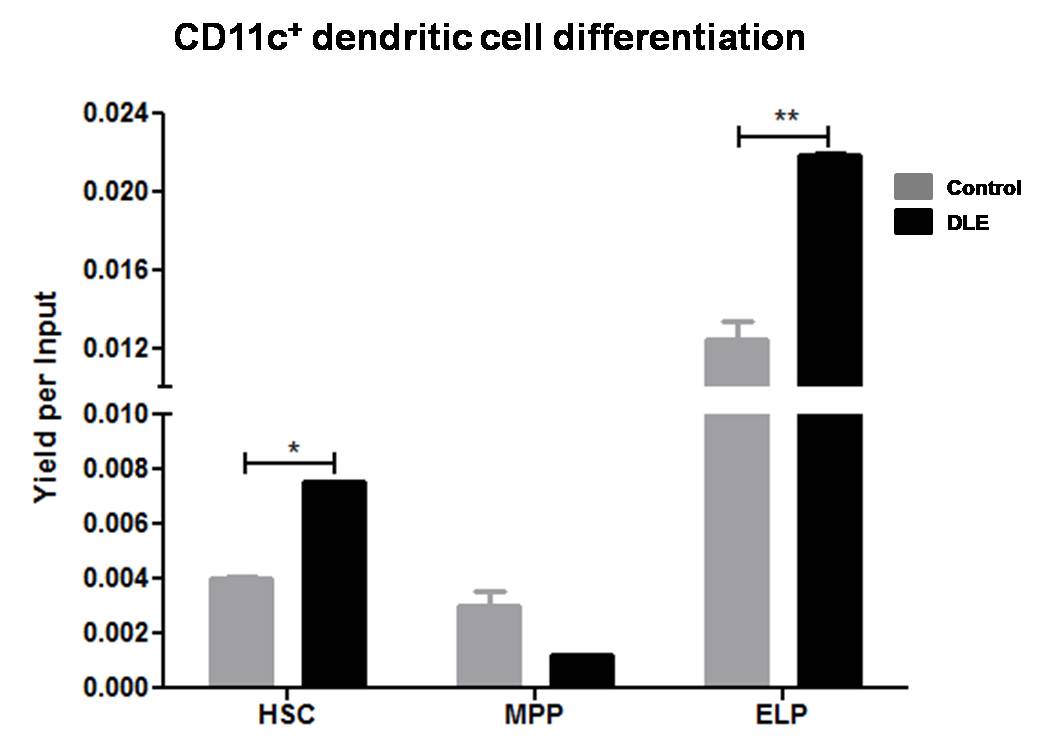


**Supplemental Figure 2. CD56^-^CD11c^+^ dendritic-like cells are differentiated from primitive stem and early lymphoid progenitors upon DLE stimulation**. Upon 24 hours of DLE Transferon^®^ stimulation, purified hematopoietic stem cells (HSC), multipotent progenitors (MPP) and early lymphoid progenitors (ELP) were co-cultured with MS-5 stromal cells for 30 days under lymphoid lineage conditions. CD56^-^CD11c^+^ DC production was investigated by flow cytometry and reported as yield per input progenitor values. For comparison, unstimulated cultures were considered as control conditions. DLE, dialyzable leukocyte extracts Transferon^®^; DC, dendritic cells.


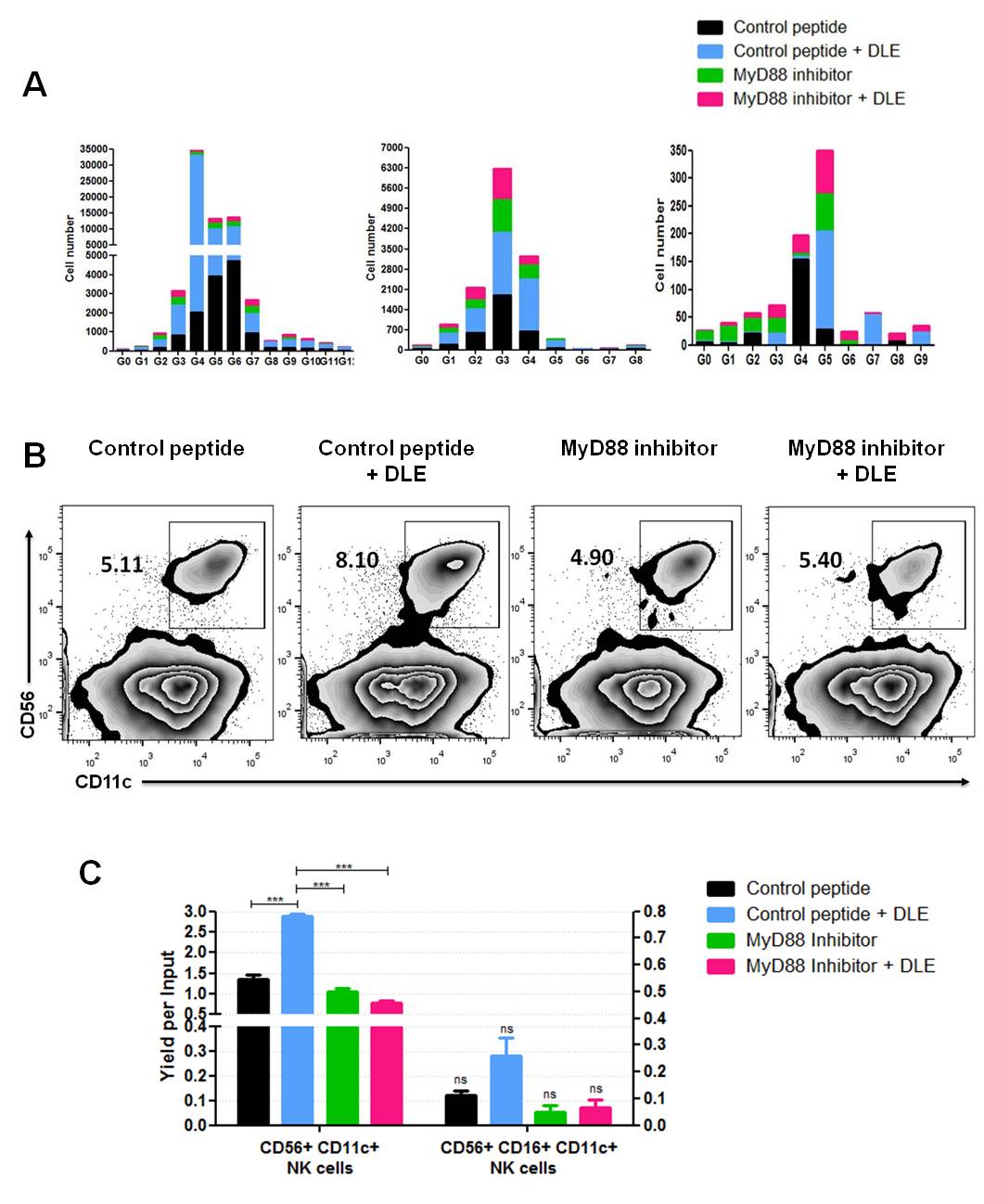


**Supplemental Figure 3. Inhibition of TLR signaling reduces proliferation and NK-differentiation potentials of DLE-stimulated CD34^+^ cells.** Umbilical cord blood (UCB) CD34^+^ cells were pre-incubated with MyD88 control peptide or with a MyD88 inhibitor peptide followed by staining with Cell Trace Violet (CTV). Cell proliferation was assessed at 72 hours by dilution of CTV (A). Cell divisions analyses (expressed as numbers of cell generations) from three independent experiments were performed using FlowJo software. For differentiation experiments, UCB CD34+ cells were pre-incubated with MyD88 control peptide or with a MyD88 inhibitor peptide for 24 hours and then co-cultured with MS-5 stromal cells for 15 days under lymphoid lineage conditions. Cell frequencies (B) and yields per input progenitor (C) of CD56^+^CD11c^+^ and CD56^+^CD16^+^CD11c^+^ NK-like cells were investigated by flow cytometry. Results from two different specimens are shown. DLE, dialyzable leukocyte extracts Transferon^®^
